# Truncated Inverted Kumaraswamy Generated Family of Distributions with Applications

**DOI:** 10.3390/e21111089

**Published:** 2019-11-07

**Authors:** Rashad A. R. Bantan, Farrukh Jamal, Christophe Chesneau, Mohammed Elgarhy

**Affiliations:** 1 Deanship of Scientific Research, King Abdulaziz University, Jeddah 21442, Saudi Arabia; rbantan@kau.edu.sa; 2 Department of Statistics, Govt. S.A Postgraduate College Dera Nawab Sahib, Bahawalpur, Punjab 63100, Pakistan; drfarrukh1982@gmail.com; 3 Department of Mathematics, Université de Caen, LMNO, Campus II, Science 3, 14032 Caen, France; 4 Valley High Institute for Management Finance and Information Systems, Obour, Qaliubia 11828, Egypt; m_elgarhy85@yahoo.com

**Keywords:** inverted Kumaraswamy distribution, truncated distribution, moments, entropy, maximum likelihood estimation, simulation, data analysis, 60E05, 62E15, 62F10

## Abstract

In this article, we introduce a new general family of distributions derived to the truncated inverted Kumaraswamy distribution (on the unit interval), called the truncated inverted Kumaraswamy generated family. Among its qualities, it is characterized with tractable functions, has the ability to enhance the flexibility of a given distribution, and demonstrates nice statistical properties, including competitive fits for various kinds of data. A particular focus is given on a special member of the family defined with the exponential distribution as baseline, offering a new three-parameter lifetime distribution. This new distribution has the advantage of having a hazard rate function allowing monotonically increasing, decreasing, and upside-down bathtub shapes. In full generality, important properties of the new family are determined, with an emphasis on the entropy (Rényi and Shannon entropy). The estimation of the model parameters is established by the maximum likelihood method. A numerical simulation study illustrates the nice performance of the obtained estimates. Two practical data sets are then analyzed. We thus prove the potential of the new model in terms of fitting, with favorable results in comparison to other modern parametric models of the literature.

## 1. Introduction

The inverted Kumaraswamy distribution was introduced by [1], with the motivation to offer a new flexible lifetime distribution with tractable distributional properties. As suggested by its name, it corresponds to the distribution of the random variable V=(1−U)/U, where *U* follows the standard Kumaraswamy distribution (more detail on the Kumaraswamy distribution can be found in the former work of [2]). Thus, it is characterized by the cumulative density function (cdf) given by

(1) $$\begin{array}{c} G_{*}\left(x;a,b\right)={\left[1-{\left(1+x\right)}^{-a}\right]}^{b},\;x>0, \end{array}$$

with a,b>0. Upon differentiation, the corresponding probability density function (pdf) is given by

(2) $$\begin{array}{c} g_{*}\left(x;a,b\right)=ab{\left(1+x\right)}^{-a-1}{\left[1-{\left(1+x\right)}^{-a}\right]}^{b-1},\;x>0, \end{array}$$

From an analytical point of view, it also corresponds to a special case of the exponentiated Lomax distribution introduced by [3] (with λ=1), having a great success in data analysis over the last decade. Having in mind the aim to explore new statistical horizons, we aim to benefit from the qualities of the inverted Kumaraswamy distribution to create a new general family of distributions. That is, we propose to truncate the inverted Kumaraswamy distribution on the unit interval and to compose it with a general cdf of a continuous distribution. Such a truncation technique has been employed with success to define new general families from well-established distributions on the semi-interval (0,+∞). See, for instance, [4] who introduced the truncated Fréchet-G family (by using the truncated Fréchet distribution on (0,1)), [5] who proposed the truncated Weibull-G family (by using the truncated Weibull distribution on (0,1)), and [6] who developed the truncated Burr-G family (by using the truncated Burr distribution on (0,1)). However, to the best of our knowledge, the consideration of the truncated inverted Kumaraswamy distribution on (0,1) in this setting remains new and motivates this study. For the mathematical foundation, the cdf of the truncated inverted Kumaraswamy distribution on (0,1) is given by

(3) $$\begin{array}{c} R\left(x;a,b\right)=\frac{G_{*}\left(x;a,b\right)-G_{*}\left(0;a,b\right)}{G_{*}\left(1;a,b\right)-G_{*}\left(0;a,b\right)}=\frac{1}{{\left(1-2^{-a}\right)}^{b}}{\left[1-{\left(1+x\right)}^{-a}\right]}^{b},\;x\in \left(0,1\right), \end{array}$$

Thus, the restriction on the support implies an adjustment on the normalization constant depending on the two shape parameters *a* and *b*, which is now ${\left(1-2^{-a}\right)}^{-b}$. Then, for any cdf of a continuous distribution G(x;ξ), by a natural composition technique, we introduce the cdf given by

(4) $$\begin{array}{c} F\left(x;a,b,\xi \right)=R\left(G\left(x;\xi \right);a,b\right)=\frac{1}{{\left(1-2^{-a}\right)}^{b}}{\left[1-{\left(1+G\left(x;\xi \right)\right)}^{-a}\right]}^{b},\;x\in R, \end{array}$$

defining the truncated inverted Kumaraswamy generated (TIK-G) family of distribution. One can notice that the TIK-G family is defined with a simple cdf, offering a tractable alternative to other families sometimes defined with sophisticated cdf. This study discusses the main distributional and practical properties of the TIK-G family, with an emphasis on entropy, as well as its potential of applicability. We also introduce a special member of the family defined with the exponential distribution as baseline, forming a new three-parameter lifetime distribution called truncated inverted Kumaraswamy exponential (TIKEx) distribution. Among its nice features, by the consideration of two practical data sets, we show that the related model has better fits to 9 other well-established models, proving the importance and interest of the TIK-G family of distribution in a data analysis setting.

The rest of the article is organized as follows. The main distributional functions related to the TIK-G family are presented in Section 2, with discussion on special members of interest. Section 3 is devoted to the mathematical properties of the TIK-G family, with a focus on the entropy. In Section 4, the maximum likelihood method is employed to obtain the estimates of the model parameters. In Section 5, we apply a special TIK model to two practical data sets, with fair comparison to other well-established models. We provide a conclusion in Section 6.

## 2. The TIK-G Family

Here, we present the main functions related to TIK-G family, a short list of special members, with discussion on the special member of the family defined with the exponential distribution as baseline.

### 2.1. Main Functions

We recall that the TIK-G family is defined by the cdf given by (4). The corresponding survival function (sf) is given by

(5) $$\begin{array}{c} S\left(x;a,b,\xi \right)=1-F\left(x;a,b,\xi \right)=1-\frac{1}{{\left(1-2^{-a}\right)}^{b}}{\left[1-{\left(1+G\left(x;\xi \right)\right)}^{-a}\right]}^{b},\;x\in R, \end{array}$$

Upon differentiation of F(x;a,b,ξ) according to *x*, the corresponding pdf is given by

(6) $$\begin{array}{c} f\left(x;a,b,\xi \right)=\frac{ab}{{\left(1-2^{-a}\right)}^{b}}g\left(x;\xi \right){\left(1+G\left(x;\xi \right)\right)}^{-a-1}{\left[1-{\left(1+G\left(x;\xi \right)\right)}^{-a}\right]}^{b-1},\;x\in R, \end{array}$$

where g(x;ξ) is the pdf corresponding to G(x;ξ).

The corresponding hazard rate function (hrf) is given by

(7) $$\begin{array}{c} h\left(x;a,b,\xi \right)=\frac{f(x;a,b,\xi )}{S(x;a,b,\xi )}=\frac{abg\left(x;\xi \right){\left(1+G\left(x;\xi \right)\right)}^{-a-1}{\left[1-{\left(1+G\left(x;\xi \right)\right)}^{-a}\right]}^{b-1}}{{\left(1-2^{-a}\right)}^{b}-{\left[1-{\left(1+G\left(x;\xi \right)\right)}^{-a}\right]}^{b}},\;x\in R, \end{array}$$

The corresponding cumulative hazard rate function (chrf) is given by

(8) $$\begin{array}{cc} H(x;a,b,\xi ) & =-\log[S(x;a,b,\xi )]\\ \; & =b\log\left(1-2^{-a}\right)-\log\left\{{\left(1-2^{-a}\right)}^{b}-{\left[1-{\left(1+G\left(x;\xi \right)\right)}^{-a}\right]}^{b}\right\},\;x\in R, \end{array}$$

The corresponding quantile function (qf), say Q(u;a,b,ξ), is characterized by the equation.

F(Q(u;a,b,ξ);a,b,ξ)=u, for u∈(0,1). After some algebra, we obtain

(9) $$\begin{array}{c} Q\left(u;a,b,\xi \right)=Q_{G}\left\{{\left[1-u^{1/b}\left(1-2^{-a}\right)\right]}^{-1/a}-1;\xi \right\},\;u\in \left(0,1\right), \end{array}$$

where $Q_{G}\left(u;\xi \right)$ denotes the qf corresponding to G(x,ξ).

Among all the important quantities related to the qf, one can mention the median defined by Med=Q(1/2;a,b,ξ) and the interquartile range defined by IQR=Q(3/4;a,b,ξ)−Q(1/4;a,b,ξ).

Upon differentiation of Q(u;a,b,ξ) according to *u*, the corresponding quantile density function (qdf) is given by

(10) $$\begin{array}{cc} q(u;a,b,\xi ) & =\frac{1}{ab}u^{1/b-1}\left(1-2^{-a}\right){\left[1-u^{1/b}\left(1-2^{-a}\right)\right]}^{-1/a-1}q_{G}\left\{{\left[1-u^{1/b}\left(1-2^{-a}\right)\right]}^{-1/a}-1;\xi \right\},\\ \; & u\in (0,1), \end{array}$$

where $q_{G}\left(u;\xi \right)$ denotes the qdf corresponding to G(x,ξ).

### 2.2. The TIKEx Distribution

The TIKEx distribution is defined by the following cdf:

(11) $$\begin{array}{c} F\left(x;a,b,\theta \right)=\frac{1}{{\left(1-2^{-a}\right)}^{b}}{\left[1-{\left(2-e^{-\theta x}\right)}^{-a}\right]}^{b},\;x>0, \end{array}$$

It corresponds to the special member of the TIK-G family defined with the cdf of the exponential distribution with parameter θ as baseline, i.e., ξ=θ and $G\left(x;\theta \right)=1-e^{-\theta x}$, x,θ>0. Then, it constitutes a new three-parameter lifetime distribution and will be the object of all the attentions in the rest of study.

**Remark** **1.** *Let us notice that, by taking a=1, the cdf given by (11) is reduced to*

(12) $$\begin{array}{c} F\left(x;1,b,\theta \right)={\left[\frac{2(1-e^{-\theta x})}{2-e^{-\theta x}}\right]}^{b},\;x>0, \end{array}$$

*which corresponds to the exponentiated cdf of the Marshall-Olkin-G family introduced by [7] defined with α=1/2 and with the the cdf of the exponential distribution with parameter θ as baseline (or the M transformation of the exponential distribution introduced by [8]). To the best of our knowledge, it is new in the literature. Hence, in our study, we consider a generalization of it thanks to the shape parameter a.*

The corresponding pdf is given by

(13) $$\begin{array}{c} f\left(x;a,b,\theta \right)=\frac{ab}{{\left(1-2^{-a}\right)}^{b}}\theta e^{-\theta x}{\left(2-e^{-\theta x}\right)}^{-a-1}{\left[1-{\left(2-e^{-\theta x}\right)}^{-a}\right]}^{b-1},\;x>0, \end{array}$$

The corresponding hrf and qf are, respectively, given by

(14) $$\begin{array}{c} h\left(x;a,b,\theta \right)=\frac{ab\theta e^{-\theta x}{\left(2-e^{-\theta x}\right)}^{-a-1}{\left[1-{\left(2-e^{-\theta x}\right)}^{-a}\right]}^{b-1}}{{\left(1-2^{-a}\right)}^{b}-{\left[1-{\left(2-e^{-\theta x}\right)}^{-a}\right]}^{b}},\;x>0, \end{array}$$

and

(15) $$\begin{array}{c} Q\left(u;a,b,\theta \right)=-\frac{1}{\theta }\log\left\{2-{\left[1-u^{1/b}\left(1-2^{-a}\right)\right]}^{-1/a}\right\},\;u\in \left(0,1\right), \end{array}$$

In particular, the median of the TIKEx distribution is given by Med=Q(1/2;a,b,θ).

**Figure 1:**
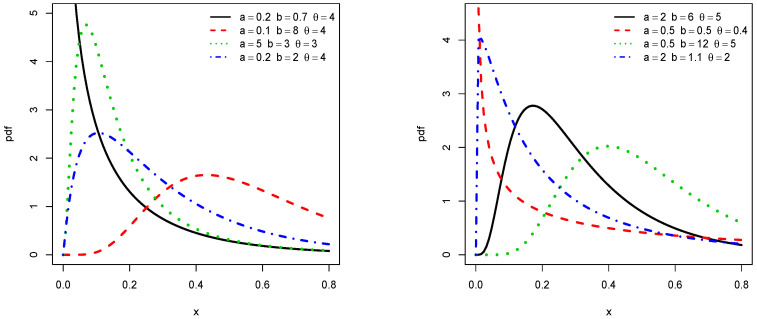
Plots of some pdfs of the truncated inverted Kumaraswamy exponential (TIKEx) distribution.

**Figure 2:**
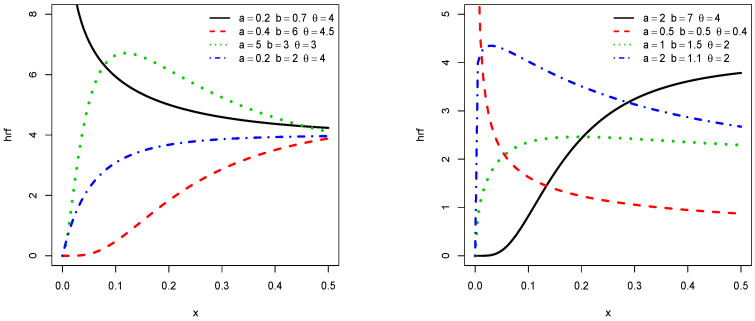
Plots of some hrfs of the TIKEx distribution.

In order to illustrate the flexibility of the shapes of f(x;a,b,θ) and h(x;a,b,θ), Figure 1 and Figure 2 display the plots of f(x;a,b,θ) and h(x;a,b,θ), respectively, for some values of the parameters *a*, *b*, and θ. We observe that the pdf is left skewed, reversed-J shaped, and approximately symmetrical, while the hrf is increasing, decreasing, upside down, and bathtub shaped.

## 3. Properties

This section is devoted to the most fundamental properties of the TIK-G family, with an emphasis on the entropy. Also, the general properties are applied to the TIKEx distribution as illustration.

### 3.1. Some Series Expansions

The following result presents a series expansion for the pdf of the TIK-G family.

**Proposition** **1.** *For any γ≥0, let us set*

(16) $$\begin{array}{c} \psi_{\gamma }\left(x;\xi \right)=\left(\gamma +1\right)g\left(x;\xi \right)S_{G}{\left(x;\xi \right)}^{\gamma }, \end{array}$$

*where $S_{G}\left(x;\xi \right)=1-G\left(x;\xi \right)$ is the sf corresponding to G(x;ξ). Then, for x such that G(x;ξ)∈(0,1), we can expand f(x;a,b,ξ) as*

(17) $$\begin{array}{c} f\left(x;a,b,\xi \right)=\sum_{k,\ell =0}^{+\infty }u_{k,\ell }\psi_{\ell }\left(x;\xi \right), \end{array}$$

*where*

(18) uk,ℓ=1(1−2−a)bbk−akℓ+1(−1)k+ℓ2−ak−ℓ−1,

*with vu=v(v−1)…(v−u+1)/u! is the generalized binomial coefficient.*

**Proof.**  The first step of the proof consists in providing a series expansion for the cdf F(x;a,b,ξ). Since G(x;ξ)∈(0,1), ${\left(1+G\left(x;\xi \right)\right)}^{-a}\in \left(0,1\right)$, $1+G\left(x;\xi \right)=2-S_{G}\left(x;\xi \right)$, and $S_{G}\left(x;\xi \right)/2\in \left(0,1\right)$, by the application of the generalized binomial formula, we can write

(19) F(x;a,b,ξ)=1(1−2−a)b1−(1+G(x;ξ))−ab=1(1−2−a)b∑k=0+∞bk(−1)k(1+G(x;ξ))−ak=1(1−2−a)b∑k,ℓ=0+∞bk−akℓ(−1)k+ℓ2−ak−ℓSG(x;ξ)ℓ,

Upon differentiation according to *x* and a change of indexes, we get the desired result. The proof of Proposition 1 is completed. □

**Remark** **2.** *By following the lines of the proof of Proposition 1, by applying the generalized binomial formula for ${\left(1+G\left(x;\xi \right)\right)}^{-ak}$ as*

(20) (1+G(x;ξ))−ak=∑ℓ=0+∞−akℓG(x;ξ)ℓ,

*we also have the following series expansion in terms of pdfs of the exponentiated-G family (see [9]):*

(21) $$\begin{array}{c} f\left(x;a,b,\xi \right)=\sum_{k,\ell =0}^{+\infty }u_{k,\ell }^{*}g_{\ell }\left(x;\xi \right), \end{array}$$

*where $g_{\ell }\left(x;\xi \right)=\left(\ell +1\right)g\left(x;\xi \right)G{\left(x;\xi \right)}^{\ell }$ and*

(22) uk,ℓ*=1(1−2−a)bbk−akℓ+1(−1)k,

Two different generalizations of Proposition 1 are given in Propositions 2 and 3.

**Proposition** **2.** *Let κ>0. Then, for x such that G(x;ξ)∈(0,1), we have the following series expansion:*

(23) $$\begin{array}{c} f\left(x;a,b,\xi \right)F{\left(x;a,b,\xi \right)}^{\kappa }=\sum_{k,\ell =0}^{+\infty }v_{k,\ell }^{\left(\kappa \right)}\psi_{\ell }\left(x;\xi \right), \end{array}$$

*where*

(24) vk,ℓ(κ)=1κ+11(1−2−a)b(κ+1)b(κ+1)k−akℓ+1(−1)k+ℓ2−ak−ℓ−1,

**Proof.**  Following the lines of the proof of Proposition 1, by replacing *b* by b(κ+1), we get

(25) F(x;a,b,ξ)κ+1=1(1−2−a)b(κ+1)1−(1+G(x;ξ))−ab(κ+1)=1(1−2−a)b(κ+1)∑k,ℓ=0+∞b(κ+1)k−akℓ(−1)k+ℓ2−ak−ℓSG(x;ξ)ℓ,

The series expansion of $f\left(x;a,b,\xi \right)F{\left(x;a,b,\xi \right)}^{\kappa }$ is obtained upon differentiation of $F{\left(x;a,b,\xi \right)}^{\kappa +1}$ according to *x* and a change of indexes. The proof of Proposition 2 is completed. □

Now, we propose an expansion for the exponentiated pdf of the TIK-G family.

**Proposition** **3.** *Let κ>0. Then, for x such that G(x;ξ)∈(0,1), we can expand $f{\left(x;a,b,\xi \right)}^{\kappa }$ as*

(26) $$\begin{array}{c} f{\left(x;a,b,\xi \right)}^{\kappa }=\sum_{k,\ell =0}^{+\infty }w_{k,\ell }^{\left(\kappa \right)}\left[g{\left(x;\xi \right)}^{\kappa -1}\psi_{\ell }\left(x;\xi \right)\right], \end{array}$$

*where*

(27) wk,ℓ(κ)=aκbκ(1−2−a)bκκ(b−1)k−ak−κ(a+1)ℓ(−1)k+ℓ2−ak−κ(a+1)−ℓ1ℓ+1,

**Proof.**  We have

(28) $$\begin{array}{c} f{\left(x;a,b,\xi \right)}^{\kappa }=\frac{a^{\kappa }b^{\kappa }}{{\left(1-2^{-a}\right)}^{b\kappa }}g{\left(x;\xi \right)}^{\kappa }{\left(1+G\left(x;\xi \right)\right)}^{-\kappa (a+1)}{\left[1-{\left(1+G\left(x;\xi \right)\right)}^{-a}\right]}^{\kappa (b-1)},\;x\in R, \end{array}$$

Since G(x;ξ)∈(0,1), ${\left(1+G\left(x;\xi \right)\right)}^{-a}\in \left(0,1\right)$, $1+G\left(x;\xi \right)=2-S_{G}\left(x;\xi \right)$, and $S_{G}\left(x;\xi \right)/2\in \left(0,1\right)$, the generalized binomial formula gives

(29) 1−(1+G(x;ξ))−aκ(b−1)=∑k=0+∞κ(b−1)k(−1)k(1+G(x;ξ))−ak,

and

(30) (1+G(x;ξ))−ak−κ(a+1)=∑ℓ=0+∞−ak−κ(a+1)ℓ2−ak−κ(a+1)−ℓ(−1)ℓSG(x;ξ)ℓ,

Therefore, by putting the above equalities together, we get the desired result. The proof of Proposition 3 is completed. □

To end this section, let us notice that, if G(x;ξ) is the cdf of the exponential distribution with parameter θ, i.e., $G\left(x;\theta \right)=1-e^{-\theta x}$, x,θ>0, then, for any positive integer *ℓ*, we have

(31) $$\begin{array}{c} \psi_{\ell }\left(x;\theta \right)=\left(\ell +1\right)g\left(x;\xi \right)S_{G}{\left(x;\xi \right)}^{\ell }=\left(\ell +1\right)\theta e^{-(\ell +1)\theta x}, \end{array}$$

which is the pdf of the exponential distribution with parameter (ℓ+1)θ. Also, for any positive real number κ, we have

(32) $$\begin{array}{c} g{\left(x;\xi \right)}^{\kappa -1}\psi_{\ell }\left(x;\theta \right)=\left(\ell +1\right)\theta^{\kappa }e^{-(\kappa +\ell )\theta x}=\frac{\ell +1}{\ell +\kappa }\theta^{\kappa -1}\psi_{\ell }^{*}\left(x;\theta \right), \end{array}$$

where $\psi_{\ell }^{*}\left(x;\theta \right)$ denotes the pdf of the exponential distribution with parameter (κ+ℓ)θ. We thus take advantage of the above results in this setting; the well-established distributional properties of the exponential distribution are useful to determine those of the TIKEx distribution.

### 3.2. Critical Points of the pdf and hrf

The critical point(s) of f(x;a,b,ξ) is/are the solution(s) of the equation ${\left\{\log\left[f(x;a,b,\xi )\right]\right\}}^{\prime }=0$ (the differentiation is according to *x*), with

(33) $$\begin{array}{cc} \log\left[f(x;a,b,\xi )\right]= & \log\left(a\right)+\log\left(b\right)-b\log\left(1-2^{-a}\right)+\log\left[g\left(x;\xi \right)\right]-\left(a+1\right)\log\left(1+G\left(x;\xi \right)\right)\\ \; & +\left(b-1\right)\log\left[1-{\left(1+G\left(x;\xi \right)\right)}^{-a}\right], \end{array}$$

implying that

(34) $$\begin{array}{c} {\left\{\log\left[f(x;a,b,\xi )\right]\right\}}^{\prime }=\frac{g{\left(x;\xi \right)}^{\prime }}{g(x;\xi )}-\left(a+1\right)\frac{g(x;\xi )}{1+G(x;\xi )}+a\left(b-1\right)\frac{g\left(x;\xi \right){\left(1+G\left(x;\xi \right)\right)}^{-a-1}}{1-{\left(1+G\left(x;\xi \right)\right)}^{-a}}, \end{array}$$

The nature of a critical point of f(x;a,b,ξ), say $x_{*}$, depends to the sign of $\lambda_{*}={\left\{\log\left[f(x;a,b,\xi )\right]\right\}}^{\prime \prime }{|}_{x=x_{*}}$. The same approach can be applied to the critical point(s) of h(x;a,b,ξ); it/they is/are given by the solution(s) of the equation ${\left\{\log\left[h(x;a,b,\xi )\right]\right\}}^{\prime }=0$, with

(35) $$\begin{array}{cc} {\left\{\log\left[h(x;a,b,\xi )\right]\right\}}^{\prime }= & \frac{g{\left(x;\xi \right)}^{\prime }}{g(x;\xi )}-\left(a+1\right)\frac{g(x;\xi )}{1+G(x;\xi )}+a\left(b-1\right)\frac{g\left(x;\xi \right){\left(1+G\left(x;\xi \right)\right)}^{-a-1}}{1-{\left(1+G\left(x;\xi \right)\right)}^{-a}}\\ \; & +ab\frac{g\left(x;\xi \right){\left(1+G\left(x;\xi \right)\right)}^{-a-1}{\left[1-{\left(1+G\left(x;\xi \right)\right)}^{-a}\right]}^{b-1}}{{\left(1-2^{-a}\right)}^{b}-{\left[1-{\left(1+G\left(x;\xi \right)\right)}^{-a}\right]}^{b}}, \end{array}$$

The nature of a critical point of h(x;a,b,ξ), say $x_{o}$, depends to the sign of $\lambda_{o}={\left\{\log\left[h(x;a,b,\xi )\right]\right\}}^{\prime \prime }{|}_{x=x_{o}}$.

In the context of the TIKEx distribution, the critical point(s) of f(x;a,b,ξ) is/are the solution(s) of the following equation according to *x*:

(36) $$\begin{array}{c} -\left(a+1\right)\frac{e^{-\theta x}}{2-e^{-\theta x}}+a\left(b-1\right)\frac{e^{-\theta x}{\left(2-e^{-\theta x}\right)}^{-a-1}}{1-{\left(2-e^{-\theta x}\right)}^{-a}}=1, \end{array}$$

and the critical point(s) of h(x;a,b,ξ) is/are the solution(s) of the following equation according to *x*:

(37) $$\begin{array}{c} -\left(a+1\right)\frac{e^{-\theta x}}{2-e^{-\theta x}}+a\left(b-1\right)\frac{e^{-\theta x}{\left(2-e^{-\theta x}\right)}^{-a-1}}{1-{\left(2-e^{-\theta x}\right)}^{-a}}+ab\frac{e^{-\theta x}{\left(2-e^{-\theta x}\right)}^{-a-1}{\left[1-{\left(2-e^{-\theta x}\right)}^{-a}\right]}^{b-1}}{{\left(1-2^{-a}\right)}^{b}-{\left[1-{\left(2-e^{-\theta x}\right)}^{-a}\right]}^{b}}=1, \end{array}$$

Clearly, the nature of a critical point depends on *a*, *b*, and θ and no close form exists. For given values of *a*, *b*, and θ, they can be determined numerically by using a mathematical software (R, Matlab, Mathematica, Python…). We refer the reader to Figure 1 and Figure 2 for a graphical illustrations of these critical points.

### 3.3. Moments

Hereafter, we consider a random variable *X* have the cdf of the TIK-G family given by (4). By assuming that it exists, for any positive integer *s*, the *s*-moment of *X* is given by

(38) $$\begin{array}{c} \mu_{s}^{\prime }=E\left(X^{s}\right)=\int_{-\infty }^{+\infty }x^{s}f\left(x;a,b,\xi \right)dx, \end{array}$$

For given G(x;ξ), *a* and *b*, we can evaluate this integral numerically. From an analytical point of view, Proposition 1 can be useful. Indeed, by assuming that the signs sum and integral can interchange, we have

(39) $$\begin{array}{c} \mu_{s}^{\prime }=\sum_{k,\ell =0}^{+\infty }u_{k,\ell }\int_{-\infty }^{+\infty }x^{s}\psi_{\ell }\left(x;\xi \right)dx, \end{array}$$

where $u_{k,\ell }$ is given by (18) and the integral term can be calculated in a simple way, depending on the complexity of G(x;ξ). For instance, in the context of the TIKEx distribution, we have ξ=θ and

(40) $$\begin{array}{c} \int_{-\infty }^{+\infty }x^{s}\psi_{\ell }\left(x;\theta \right)dx=\int_{0}^{+\infty }x^{s}\left(\ell +1\right)\theta e^{-(\ell +1)\theta x}dx=\frac{s!}{{\left(\ell +1\right)}^{s}\theta^{s}}, \end{array}$$

Hence, in this special case, we have

(41) $$\begin{array}{c} \mu_{s}^{\prime }=\frac{s!}{\theta^{s}}\sum_{k,\ell =0}^{+\infty }u_{k,\ell }\frac{1}{{\left(\ell +1\right)}^{s}}, \end{array}$$

The mean of *X* is given by $\mu =\mu_{1}^{\prime }=E\left(X\right)$ and the variance of *X* is given by $\sigma^{2}=E\left[{\left(X-\mu \right)}^{2}\right]=\mu_{2}^{\prime }-\mu^{2}$. More generally, the *s*-th central moment of *X*, i.e., $\mu_{s}=E\left[{\left(X-\mu \right)}^{s}\right]$, can be deduced via the binomial formula such as

(42) μs=∑k=0ssk(−1)kμkμs−k′orμs=∑k=0ssk(−1)s−kμs−kμk′,

Also, the *s*-th general coefficient of *X* is given by

(43) $$\begin{array}{c} C_{s}=\frac{\mu_{s}}{\sigma^{s}}, \end{array}$$

For s=1, we obtain $C_{1}$, which is used to define the coefficient of variation of *X* as

(44) $$\begin{array}{c} CV=\frac{1}{C_{1}}, \end{array}$$

This coefficient is an useful standardized measure of dispersion. For s=3, $C_{s}$ becomes the skewness coefficient of *X* and for s=4, it becomes the kurtosis coefficients of *X*, which are traditionally used to evaluate the asymmetry and the peakedness of the corresponding distribution, respectively.

We end this subsection by giving numerical values of some central, dispersion, skewness, and kurtosis parameters for the TIKEx distribution in Table 1.

From Table 1, for the considered values of the parameters, we see that the TIKEx distribution has varying median, mean, and dispersion (with CV ∈[0.89,2]). Also, it is mainly right skewed (with $C_{3}\in \left[2,5.9\right]$) and shows high variation for the kurtosis coefficient (with $C_{4}\in \left[12,112\right]$).

### 3.4. Probability Weighted Moments

By assuming that it exists, for any positive integers *s* and *t*, the (s,t)-probability weighted moment of *X* is given by

(45) $$\begin{array}{c} \mu_{s,t}^{\prime }=E\left[X^{s}F{\left(X;a,b,\xi \right)}^{t}\right]=\int_{-\infty }^{+\infty }x^{s}f\left(x;a,b,\xi \right)F{\left(x;a,b,\xi \right)}^{t}dx, \end{array}$$

Again, for given G(x;ξ), *a* and *b*, this integral can be evaluated numerically. One can also use Proposition 2 in the following manner. By assuming that the signs sum and integral can interchange, we have

(46) $$\begin{array}{c} \mu_{s,t}^{\prime }=\sum_{k,\ell =0}^{+\infty }v_{k,\ell }^{\left(t\right)}\int_{-\infty }^{+\infty }x^{s}\psi_{\ell }\left(x;\xi \right)dx, \end{array}$$

where $v_{k,\ell }^{\left(t\right)}$ is given by (24). In the context of the TIKEx distribution, we have

(47) $$\begin{array}{c} \mu_{s,t}^{\prime }=\frac{s!}{\theta^{s}}\sum_{k,\ell =0}^{+\infty }v_{k,\ell }^{\left(t\right)}\frac{1}{{\left(\ell +1\right)}^{s}}, \end{array}$$

The probability weighted moments appear naturally in many applied areas, as those using order statistics. We refer the reader to [10].

### 3.5. Incomplete Moments

By assuming that it exists, for any positive integers *s*, the *s*-incomplete moment of *X* is given by

(48) $$\begin{array}{c} \mu_{s}^{\prime }\left(y\right)=E\left(X^{s}Y_{y}\right)=\int_{-\infty }^{y}x^{s}f\left(x;a,b,\xi \right)dx, \end{array}$$

where $Y_{y}$ is a random variable such that $Y_{y}=X$ if X≤y and $Y_{y}=0$ elsewhere. Owing to Proposition 1, we can express $\mu_{s}^{\prime }\left(y\right)$ as

(49) $$\begin{array}{c} \mu_{s}^{\prime }\left(y\right)=\sum_{k,\ell =0}^{+\infty }u_{k,\ell }\int_{-\infty }^{y}x^{s}\psi_{\ell }\left(x;\xi \right)dx, \end{array}$$

For the special case of the TIKEx distribution, we have

(50) $$\begin{array}{c} \mu_{s}^{\prime }\left(y\right)=\frac{1}{\theta^{s}}\sum_{k,\ell =0}^{+\infty }u_{k,\ell }\frac{1}{{\left(\ell +1\right)}^{s}}\gamma \left(s+1,\left(\ell +1\right)\theta y\right), \end{array}$$

where $\gamma \left(a,x\right)=\int_{0}^{x}y^{a-1}e^{-y}dy$ is the lower incomplete gamma function.

Among the possible applications of the incomplete moments, we would like to mention the Lorenz and Bonferroni curves using the first incomplete moment; they are, respectively, defined by

(51) $$\begin{array}{c} L\left(\pi \right)=\frac{\mu_{1}^{\prime }\left[Q\left(\pi ;a,b,\xi \right)\right]}{\mu },\;B\left(\pi \right)=\frac{\mu_{1}^{\prime }\left[Q\left(\pi ;a,b,\xi \right)\right]}{\pi \mu },\;\pi \in \left(0,1\right), \end{array}$$

**Figure 3:**
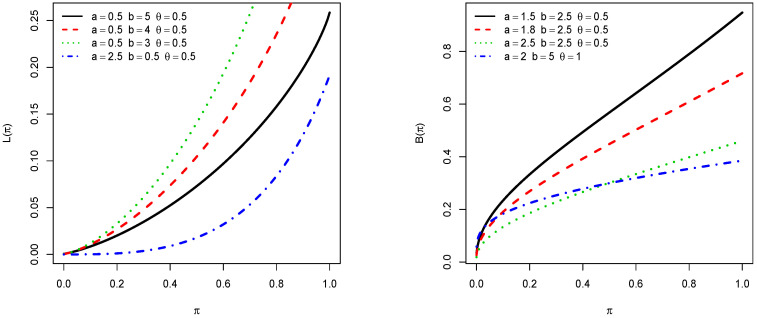
Plots of the Lorenz and Bonferroni curves of the TIKEx distribution.

Numerous real life applications employed such curves. We refer the reader to [11] and [12], respectively. Figure 3 shows the plots of these curves in the context of the TIKEx distribution for selected values of parameters.

### 3.6. Entropy

The entropy of a random variable *X* is a measure of variation of the uncertainty: high entropy means high uncertainty. The entropy plays a fundamental role in information theory, where several entropy measures have been introduced. We refer the reader to the review of [13], and the references therein. This subsection is devoted to two notable ones: the Rényi entropy and Shannon entropy.

#### 3.6.1. Rényi Entropy

The Rényi entropy of *X* is defined by

(52) $$\begin{array}{c} I_{\delta }=\frac{1}{1-\delta }\log\left\{\int_{-\infty }^{+\infty }f{\left(x;a,b,\xi \right)}^{\delta }dx\right\}, \end{array}$$

where δ>0 and δ≠1. The former work and motivations can be found in [14]. Under some configuration on G(x;ξ), *a*, *b*, and δ, it can be computed numerically. Also, owing to Proposition 3, we can express $I_{\delta }$ as

(53) $$\begin{array}{c} I_{\delta }=\frac{1}{1-\delta }\log\left\{\sum_{k,\ell =0}^{+\infty }w_{k,\ell }^{\left(\delta \right)}\int_{-\infty }^{+\infty }g{\left(x;\xi \right)}^{\delta -1}\psi_{\ell }\left(x;\xi \right)dx\right\}, \end{array}$$

where $w_{k,\ell }^{\left(\delta \right)}$ is defined by (27). As example, for the TIKEx distribution, by using (32), we have

(54) $$\begin{array}{c} \int_{-\infty }^{+\infty }g{\left(x;\theta \right)}^{\delta -1}\psi_{\ell }\left(x;\theta \right)dx=\frac{\ell +1}{\ell +\delta }\theta^{\delta -1}\int_{0}^{+\infty }\psi_{\ell }^{*}\left(x;\theta \right)dx=\frac{\ell +1}{\ell +\delta }\theta^{\delta -1}, \end{array}$$

implying that

(55) $$\begin{array}{c} I_{\delta }=-\log\left(\theta \right)+\frac{1}{1-\delta }\log\left\{\sum_{k,\ell =0}^{+\infty }w_{k,\ell }^{\left(\delta \right)}\frac{\ell +1}{\ell +\delta }\right\}, \end{array}$$

Numerical values of the Rényi entropy for the TIKEx distribution for various values of the parameters are documented in Table 2.

In Table 2, we observe that the Rényi entropy can take negative and positive values belonging to the interval [−6.13,1.66] for the considered values of δ, *a*, *b*, and θ. Thus, these parameters have a strong effect on the Rényi entropy, showing different degrees of uncertainty.

#### 3.6.2. Shannon Entropy

The Shannon entropy of *X* is defined by

(56) $$\begin{array}{c} \eta =-E\left\{\log\left[f\left(X;a,b,\xi \right)\right]\right\}=-\int_{-\infty }^{+\infty }\log\left[f\left(x;a,b,\xi \right)\right]f\left(x;a,b,\xi \right)dx, \end{array}$$

It has been introduced by [15]. One can show that it is obtained by applying δ→1 to the Rényi entropy presented above. Another expression comes from the former definition. Indeed, by using the expectation expression, we can write

(57) $$\begin{array}{cc} \eta = & -\log\left(a\right)-\log\left(b\right)+b\log\left(1-2^{-a}\right)-E\left\{\log[g(X;\xi )]\right\}+\left(a+1\right)E\left\{\log[1+G(X;\xi )]\right\}\\ \; & -\left(b-1\right)E\left\{\log\left[1-{\left(1+G\left(X;\xi \right)\right)}^{-a}\right]\right\}, \end{array}$$

We now propose a series expansion for η. Owing to Proposition 1, we have

(58) $$\begin{array}{c} E\left\{\log\left[g\left(X;\xi \right)\right]\right\}=\sum_{k,\ell =0}^{+\infty }u_{k,\ell }\int_{-\infty }^{+\infty }\log\left[g\left(x;\xi \right)\right]\psi_{\ell }\left(x;\xi \right)dx, \end{array}$$

Under some circumstance, the integral term can be determined. On the other hand, the series expansion of the logarithmic function gives

(59) $$\begin{array}{c} E\left\{\log\left[1+G\left(X;\xi \right)\right]\right\}=\sum_{k=1}^{+\infty }\frac{{\left(-1\right)}^{k+1}}{k}E\left[G{\left(X;\xi \right)}^{k}\right], \end{array}$$

and, with the application of the generalized binomial formula in a second step,

(60) Elog1−(1+G(X;ξ))−a=−∑k=1+∞1kE(1+G(X;ξ))−ak=−∑k=1+∞∑ℓ=0+∞−akℓ1kEG(X;ξ)ℓ,

For the expectations terms in the sums, one can notice that, for any positive integer κ, by Remark 2,

(61) $$\begin{array}{c} E\left[G{\left(X;\xi \right)}^{\kappa }\right]=\sum_{k,\ell =0}^{+\infty }u_{k,\ell }^{*}\int_{-\infty }^{+\infty }G{\left(x;\xi \right)}^{\kappa }g_{\ell }\left(x;\xi \right)dx=\sum_{k,\ell =0}^{+\infty }u_{k,\ell }^{*}\frac{\ell +1}{\ell +\kappa +1}, \end{array}$$

(one can also use Proposition 1, but with more developments in this case). 

Some numerical values Shannon entropy for some values of the parameters are collected in Table 3.

For the considered values of *a*, *b*, and θ in Table 3, the Shannon entropy takes its values into the interval [−0.95,1.9]. Thus, the amount of uncertainty is impacted by these parameters, showing the richness of the TIKEx distribution in this sense.

## 4. Maximum Likelihood Estimation

This section focuses on the estimation of the TIK-G model parameters by the maximum likelihood method.

### 4.1. Basics on the Maximum Likelihood Method

Let $x_{1},\ldots ,x_{n}$ be a random sample of size *n* of *X*. Then, the log-likelihood functions is defined by

(62) $$\begin{array}{cc} \ell (a,b,\xi ) & =\sum_{i=1}^{n}\log\left[f(x_{i};a,b,\xi )\right]=n\log\left(a\right)+n\log\left(b\right)-nb\log\left(1-2^{-a}\right)+\sum_{i=1}^{n}\log\left[g\left(x_{i};\xi \right)\right]\\ \; & -\left(a+1\right)\sum_{i=1}^{n}\log\left(1+G\left(x_{i};\xi \right)\right)+\left(b-1\right)\sum_{i=1}^{n}\log\left[1-{\left(1+G\left(x_{i};\xi \right)\right)}^{-a}\right], \end{array}$$

Assuming that ℓ(a,b,ξ) is differentiable according to *a*, *b*, and ξ, the maximum likelihood estimates (MLEs) are given by the simultaneous solutions of the following equations: ∂ℓ(a,b,ξ)/∂a=0, ∂ℓ(a,b,ξ)/∂b=0 and ∂ℓ(a,b,ξ)/∂ξ=0, where

(63) $$\begin{array}{c} \frac{\partial }{\partial a}\ell \left(a,b,\xi \right)=\frac{n}{a}-nb\frac{\log\left(2\right)2^{-a}}{1-2^{-a}}-\sum_{i=1}^{n}\log\left(1+G\left(x_{i};\xi \right)\right)+\left(b-1\right)\sum_{i=1}^{n}\frac{{\left(1+G\left(x_{i};\xi \right)\right)}^{-a}\log\left(1+G\left(x_{i};\xi \right)\right)}{1-{\left(1+G\left(x_{i};\xi \right)\right)}^{-a}}, \end{array}$$

(64) $$\begin{array}{c} \frac{\partial }{\partial b}\ell \left(a,b,\xi \right)=\frac{n}{b}-n\log\left(1-2^{-a}\right)+\sum_{i=1}^{n}\log\left[1-{\left(1+G\left(x_{i};\xi \right)\right)}^{-a}\right], \end{array}$$

and, by setting $g_{\xi }\left(x;\xi \right)=\partial g\left(x;\xi \right)/\partial \xi$ and $G_{\xi }\left(x;\xi \right)=\partial G\left(x;\xi \right)/\partial \xi$,

(65) $$\begin{array}{c} \frac{\partial }{\partial \xi }\ell \left(a,b,\xi \right)=\sum_{i=1}^{n}\frac{g_{\xi }\left(x_{i};\xi \right)}{g(x_{i};\xi )}-\left(a+1\right)\sum_{i=1}^{n}\frac{G_{\xi }\left(x_{i};\xi \right)}{1+G(x_{i};\xi )}+\left(b-1\right)a\sum_{i=1}^{n}\frac{G_{\xi }\left(x_{i};\xi \right){\left(1+G\left(x_{i};\xi \right)\right)}^{-a-1}}{1-{\left(1+G\left(x_{i};\xi \right)\right)}^{-a}}, \end{array}$$

Let us denote the MLES of *a*, *b*, and ξ by $\hat{a}$, $\hat{b}$, and $\hat{\xi }$, respectively. Then, it follows from the equation $\partial \ell (\hat{a},\hat{b},\hat{\xi })/\partial b=0$ the following simple relation:

(66) $$\begin{array}{c} \hat{b}={\left\{\log\left(1-2^{-\hat{a}}\right)-\frac{1}{n}\sum_{i=1}^{n}\log\left[1-{\left(1+G\left(x_{i};\hat{\xi }\right)\right)}^{-\hat{a}}\right]\right\}}^{-1}, \end{array}$$

Under standard regularity conditions, all the well-established theoretical properties behind the MLEs can be applied, allowing the construction of confidence interval and statistical tests, among others. The complete theory can be found in [16].

To end this subsection, we would like to mention that, in the context of the TIKEx distribution, i.e., ξ=θ and $G\left(x;\theta \right)=1-e^{-\theta x}$, x,θ>0, the above partial differential becomes

(67) $$\begin{array}{cc} \frac{\partial }{\partial a}\ell \left(a,b,\theta \right) & =\frac{n}{a}-nb\frac{\log\left(2\right)2^{-a}}{1-2^{-a}}-\sum_{i=1}^{n}\log\left(2-e^{-\theta x_{i}}\right)+\left(b-1\right)\sum_{i=1}^{n}\frac{{\left(2-e^{-\theta x_{i}}\right)}^{-a}\log\left(2-e^{-\theta x_{i}}\right)}{1-{\left(2-e^{-\theta x_{i}}\right)}^{-a}}, \end{array}$$

(68) $$\begin{array}{cc} \frac{\partial }{\partial b}\ell \left(a,b,\theta \right) & =\frac{n}{b}-n\log\left(1-2^{-a}\right)+\sum_{i=1}^{n}\log\left[1-{\left(2-e^{-\theta x_{i}}\right)}^{-a}\right], \end{array}$$

and

(69) $$\begin{array}{cc} \frac{\partial }{\partial \theta }\ell \left(a,b,\theta \right) & =\frac{n}{\theta }-\sum_{i=1}^{n}x_{i}-\left(a+1\right)\sum_{i=1}^{n}\frac{x_{i}e^{-\theta x_{i}}}{2-e^{-\theta x_{i}}}+\left(b-1\right)a\sum_{i=1}^{n}\frac{x_{i}e^{-\theta x_{i}}{\left(2-e^{-\theta x_{i}}\right)}^{-a-1}}{1-{\left(2-e^{-\theta x_{i}}\right)}^{-a}}, \end{array}$$

### 4.2. Simulation

Here, we consider exclusively the TIKEx model. Let *X* be a random variable following the TIKEx distribution with parameters *a*, *b*, and θ. We simulate values from *X* and, for each n=50, 100, 200, 500, and 1000, we consider N=1000 random samples of size *n* from *X*. This simulation is based on the fact that, for any random variable *A* following the uniform distribution U(0,1), $x_{A}=Q\left(A;a,b,\theta \right)$ following the TIKEx distribution with parameters *a*, *b*, and θ. We consider 8 sets of different parameters with *b* fixed as b=2. Then, the performance of the MLEs is evaluated by considering the mean of the estimates (estimate) and the root-mean-squared error (RMSE), respectively defined by

(70) $$\begin{array}{c} {\mathrm{Estimate}}_{\phi }=\frac{1}{N}\sum_{i=1}^{N}{\hat{\phi }}_{i},\;{\mathrm{RMSE}}_{\phi }=\sqrt{\frac{1}{N}\sum_{i=1}^{N}{\left({\hat{\phi }}_{i}-\phi \right)}^{2}}, \end{array}$$

where ϕ denotes *a* or *b* or θ and ${\hat{\phi }}_{i}$ denotes the corresponding MLE obtained by using the *i*-th random sample. The numerical results, obtained by the use of the R software, are documented in Table 4.

From Table 4, we see that the RMSEs of the model parameters decrease as *n* increases, which is consistent with the maximum likelihood method theory (see [16]).

## 5. Applications

This section provides an application to show how the TIKEx distribution can be applied in practice. With this aim in mind, we compare the TIKEx model with those of the Weibull-exponential (WEx) model (see [17]), Lomax-exponential (LEx) model (see [18]), gamma-exponentiated exponential (GEx) model (see [19]), Burr III-exponential (BrEx) model (see [20]), Burr X-exponential (BXEx) model (see [21]), standard exponential (Ex) model, standard two-parameter Weibull (W2) model, three-parameter Weibull (W3) model (see [22]), and standard log-normal (LN) model.

The first data set The first data set is given by [23]. The data refers to the time between failures for repairable items. The data are: 1.43, 0.11, 0.71, 0.77, 2.63, 1.49, 3.46, 2.46, 0.59, 0.74, 1.23, 0.94, 4.36, 0.40, 1.74, 4.73, 2.23, 0.45, 0.70, 1.06, 1.46, 0.30, 1.82, 2.37, 0.63, 1.23, 1.24, 1.97, 1.86, 1.17.

The second data set The second data set consists of 179 values of successive failure of the air conditioning system. For the data and more detail, we refer the reader to [24,25].

**Figure 4:**
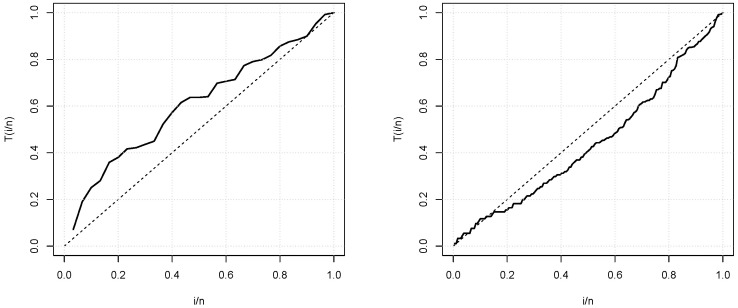
Total test time (TTT) plots for the first and second data sets, respectively.

First of all, we would like to mention that the coming data analyzes are performed by the use of the R software. Table 5 shows first description of the data, revealing different natures, mainly on the range, the skewness and kurtosis. Figure 4 presents the total test time (TTT) plots for the two data sets. We can see that the curve in the first TTT plot is concave which indicates that the first data set is related to an increasing failure rate. The curve in the second TTT plot is convex, indicating that the second data set is related to a decreasing failure rate (see [26] for further detail on TTT plot). These cases are covered by the TIKEx distribution, motivating its used (see Figure 2).

The MLEs of the model parameters are considered, the essential of the MLEs for the TIKEx model can be found in the Section 4.1. For the first data set, Table 6 presents the MLEs for all the considered models. Then, Table 7 presents some standard goodness-of-fit measures, including the AIC: Akaike Information Criterion, BIC: Bayesian Information Criterion, A${}^{*}$: Anderson–Darling statistic and W${}^{*}$: Cramer–von Mises statistic. Also, the minus log-likelihood for the estimated model is computed ($-\hat{\ell }$). The lower the values of these measures, the better the fit. We complete them by providing the KS: Kolmogorov–Smirnov statistic, along with its *p*-value.

For the second data sets, Table 8 gives the MLEs for all the considered models. Table 9 is the analogue of Table 7 but for the second data set.

**Figure 5:**
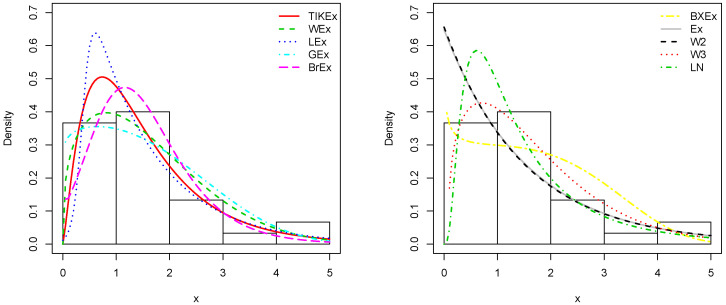
Estimated pdfs for the first data set.

**Figure 6:**
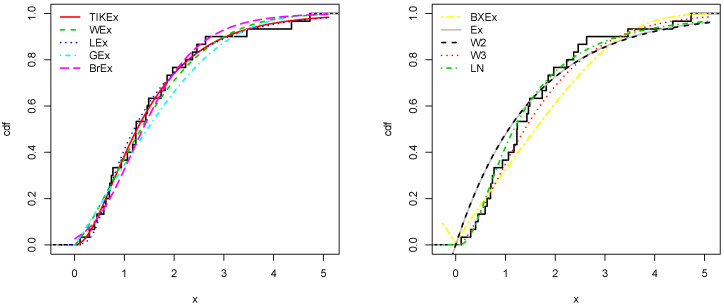
Estimated cdfs for the first data set.

**Figure 7:**
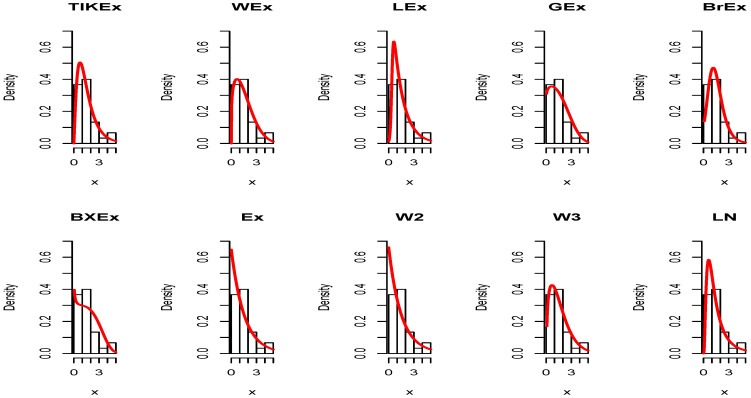
Estimated pdfs of the models for the first data set.

**Figure 8:**
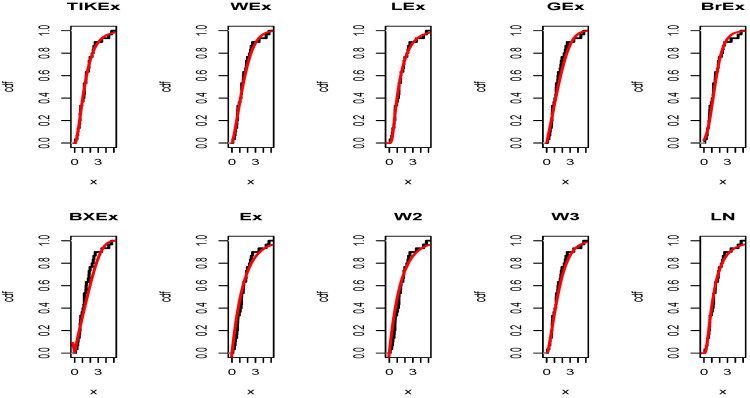
Estimated cdfs of the models for the first data set.

For the data set 1, all the estimated pdfs, superposed on the related histogram of the data, are given in Figure 5. A simultaneous comparison of the estimated cdfs with the empirical cdf of the data can be seen in Figure 6. Figure 7 and Figure 8 are the same of Figure 5 and Figure 6, respectively, but with an individual treatment of the estimated functions.

**Figure 9:**
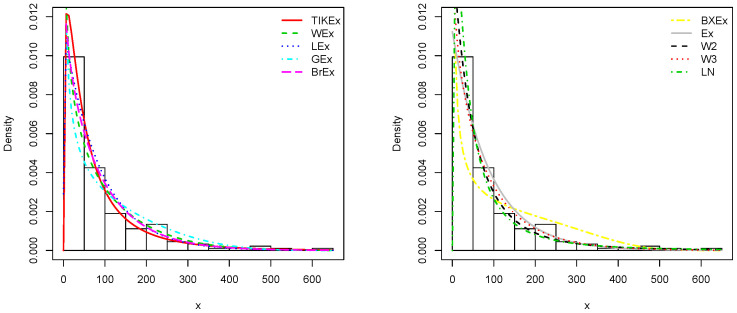
Estimated pdfs for the second data set.

**Figure 10:**
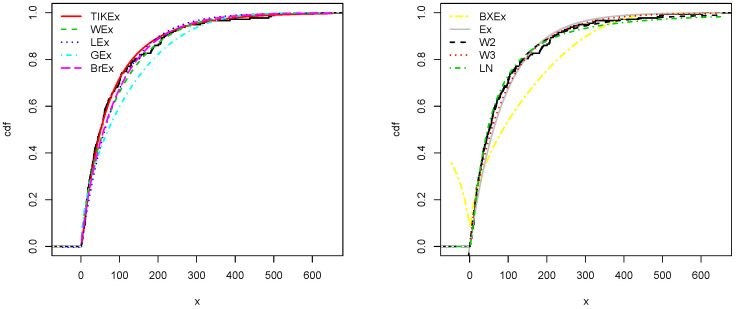
Estimated cdfs for the second data set.

**Figure 11:**
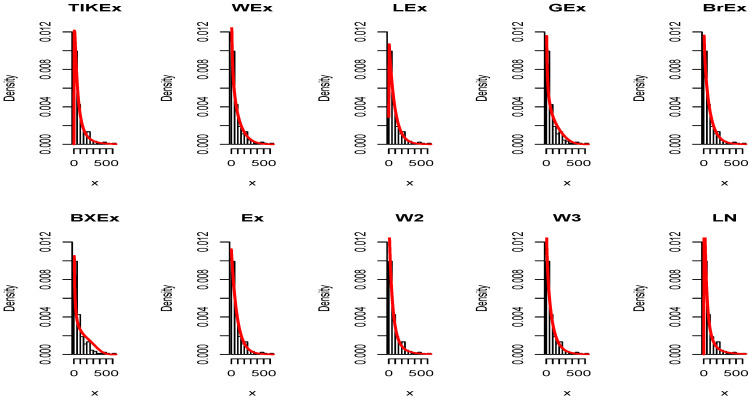
Estimated pdfs of the models for the second data set.

**Figure 12:**
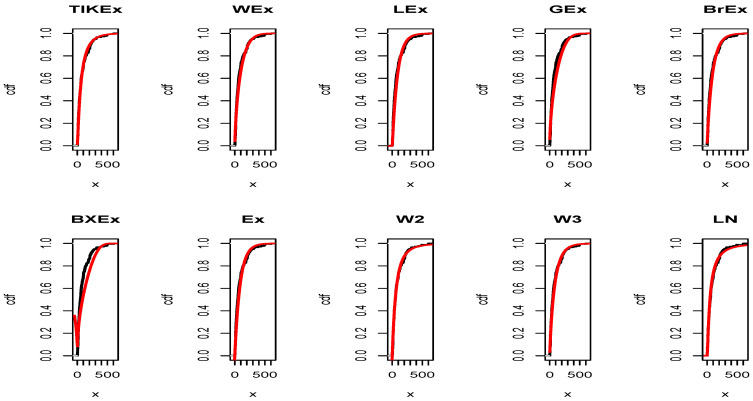
Estimated cdfs of the models for the second data set.

Similarly, for the data set 2, the estimated pdfs and cdfs can be observed in Figure 9 and Figure 10, respectively. Figure 11 and Figure 12 propose the same, respectively, but with an individual treatment of the estimated functions.

In view of Table 7 and Table 9, the TIKEx model is the best (smallest AIC, smallest BIC…), except for the second data set where the log-normal model has a better KS and *p*-value (but the TIKEx model remains the best for the other measures). The superiority of the TIKEx model is also supported by all the figures illustrating the fitting of the models over the considered data. All these results motivate the importance of the TIKEx model in the context of data analysis.

## 6. Conclusions

A new general family of distributions is introduced by the use of a truncated version of the inverted Kumaraswamy distribution and composition. It is called the TIK-G family. Thanks to its simplicity, richness, and nice flexible properties demonstrated along the study, it provides a suitable alternative to other general families somehow complex. Its main theoretical properties are discussed, with expressions and numerical analyzes for the Rényi entropy and Shannon entropy. A special focus is put on the special member of the family defined with the exponential distribution, called the TIKEx distribution. After showing its undeniable qualities (flexible pdf and hrf, series expansions for the moments, entropy…), we prove that the TIKEx model is capable of fitting various types of data, better than several modern models also derived to the exponential distribution. Thus, we hope that the TIK-G family can attract wider applications in many applied field where a sharp data analysis is essential to explain new phenomena.

## Figures and Tables

**Table 1:** The numerical values of the central parameters (Med and μ), some first moments ($\mu_{2}^{\prime }$, $\mu_{3}^{\prime }$ and $\mu_{4}^{\prime }$), variance $\sigma^{2}$, skewness ($C_{3}$), kurtosis ($C_{4}$), and coefficient of variation (CV) of the TIKEx distribution for some parameter values.

(*a*, *b*, θ)	Med	μ	$\mu_{2}^{\prime }$	$\mu_{3}^{\prime }$	$\mu_{4}^{\prime }$	$\sigma^{2}$	$C_{3}$	$C_{4}$	CV
(0.5, 0.5, 0.5)	0.7309	0.9175	2.8803	15.6847	120.2783	2.0384	3.1958	19.4201	1.5559
(1.5, 0.5, 0.5)	0.1954	0.7433	2.1122	11.0235	83.0045	1.5596	3.6627	24.8965	1.6800
(2.5, 0.5, 0.5)	0.1128	0.5980	1.5117	7.4977	55.2596	1.1541	4.2044	32.3790	1.7964
(5.0, 0.5, 0.5)	0.0548	0.3507	0.6128	2.5498	17.4948	0.4898	5.8093	63.5770	1.9955
(0.5, 1.0, 0.5)	3.0081	1.5291	5.3943	30.5369	237.7203	3.0561	2.4223	12.5090	1.1432
(0.5, 0.5, 1.0)	0.3654	0.4587	0.7200	1.9605	7.5173	0.5096	3.1958	23.2365	1.5559
(1.0, 0.5, 1.0)	0.1541	0.4134	0.6187	1.6507	6.2740	0.4477	3.4198	25.7699	1.6184
(1.0, 0.5, 2.0)	0.0770	0.2067	0.1546	0.2063	0.3921	0.1119	3.4198	33.4255	1.6184
(5.0, 0.5, 2.0)	0.0137	0.0876	0.03830	0.0398	0.0683	0.0306	5.8093	79.7019	1.9955
(5.0, 0.5, 5.0)	0.0054	0.0350	0.0061	0.0025	0.0017	0.0048	5.8093	111.9517	1.9955
(5.0, 1.0, 5.0)	0.0122	0.0598	0.0117	0.0050	0.0034	0.0082	4.5217	97.2774	1.5136
(5.0, 3.0, 5.0)	0.0225	0.1229	0.0314	0.0146	0.0103	0.0163	3.2159	100.9527	1.0407
(5.0, 5.0, 5.0)	0.0258	0.1637	0.0482	0.0237	0.01707	0.0214	2.7876	111.0229	0.8945

**Table 2:** Values of the Rényi entropy of the TIKEx distribution for some parameter values.

δ	*a*	*b*	θ	Rényi Entropy
0.5	0.5	0.5	0.5	−0.76558
0.5	1.0	0.5	0.5	−0.7256
0.5	5.0	0.5	0.5	−0.37040
0.5	5.0	1.0	0.5	−0.5772
0.5	5.0	5.0	0.5	−0.9030
0.5	5.0	5.0	1.0	−0.5564
0.5	5.0	5.0	5.0	0.2482
0.5	0.5	0.5	5.0	0.3857
0.5	2.0	0.5	5.0	0.5099
0.5	2.0	0.5	5.0	0.7808
2.0	2.0	1.0	0.5	0.6079
2.0	2.0	2.0	0.5	1.2298
2.0	2.0	5.0	0.5	1.6593
2.0	5.0	5.0	0.5	1.0207
2.0	5.0	5.0	2.0	−0.3655
2.0	5.0	5.0	5.0	−1.2818
5.0	5.0	5.0	1.0	0.3039
5.0	5.0	5.0	5.0	−6.1337

**Table 3:** Values of the Shannon entropy of the TIKEx distribution for some parameter values.

*a*	*b*	θ	Shannon Entropy
2.0	5.0	0.5	1.8957
2.0	5.0	1.0	1.2025
2.0	5.0	3.0	0.1039
2.0	5.0	5.0	−0.4068
0.5	1.0	0.5	1.4128
1.0	1.0	0.5	1.3068
3.0	1.0	0.5	0.8544
5.0	1.0	0.5	0.4170
5.0	2.0	0.5	0.9045
5.0	5.0	0.5	1.3442
5.0	5.0	1.0	0.6510
5.0	5.0	3.0	−0.4475
5.0	5.0	5.0	−0.9583

**Table 4:** Simulations of the TIKEx model parameters with the maximum likelihood method for different sets of values in order (a,θ,b) and fixed b=2.

	**Set 1:**	(0.5, 0.5, 2)	**Set 2:**	(1.5, 0.5, 2)	**Set 3:**	(2, 0.5, 2)	**Set 4:**	(1.5, 1.5, 2)
*n*	**Estimates**	**RMSEs**	**Estimates**	**RMSEs**	**Estimates**	**RMSEs**	**Estimates**	**RMSEs**
50	0.872	1.632	1.029	1.780	1.213	2.046	1.213	2.160
	0.485	0.122	0.563	0.157	0.595	0.202	1.535	0.442
	2.201	0.716	2.099	0.962	2.015	0.624	1.988	0.789
100	0.794	1.093	1.202	1.384	1.475	1.486	1.533	1.291
	0.490	0.092	0.525	0.117	0.562	0.150	1.562	0.333
	2.164	0.499	1.970	0.374	1.973	0.443	2.129	0.501
200	0.670	0.901	1.360	1.248	1.477	1.224	1.385	1.205
	0.497	0.073	0.513	0.104	0.535	0.097	1.513	0.275
	2.087	0.335	1.994	0.286	1.894	0.315	1.975	0.367
500	0.419	0.609	1.098	0.996	1.885	0.936	1.225	0.910
	0.503	0.042	0.524	0.073	0.506	0.072	1.549	0.186
	1.981	0.222	1.895	0.249	1.959	0.221	1.946	0.262
1000	0.291	0.447	1.080	0.858	1.712	0.832	0.943	0.837
	0.510	0.030	0.525	0.056	0.520	0.066	1.594	0.158
	1.949	0.145	1.905	0.248	1.940	0.189	1.868	0.222
	**Set 5:**	(0.5, 1.5, 2)	**Set 6:**	(2, 1.5, 2)	**Set 7:**	(2, 2, 2)	**Set 8:**	(1.5, 2, 2)
*n*	**Estimates**	**RMSEs**	**Estimates**	**RMSEs**	**Estimates**	**RMSEs**	**Estimates**	**RMSEs**
50	1.020	2.135	1.869	1.913	1.829	2.128	1.329	2.259
	1.514	0.409	1.609	0.492	2.155	0.703	2.094	0.641
	2.425	1.204	2.041	0.559	2.144	0.839	2.060	0.607
100	1.056	1.303	1.747	1.598	2.403	1.607	1.617	1.389
	1.451	0.275	1.622	0.421	1.965	0.512	2.047	0.454
	2.259	0.555	2.002	0.442	2.138	0.470	2.154	0.457
200	0.621	0.887	1.840	1.218	1.778	1.061	1.340	1.004
	1.487	0.218	1.573	0.296	2.089	0.387	2.059	0.339
	2.052	0.321	2.009	0.345	1.980	0.303	1.992	0.314
500	0.645	0.616	1.744	0.852	2.015	0.811	1.449	0.714
	1.484	0.125	1.560	0.192	2.005	0.272	2.031	0.240
	2.069	0.259	1.968	0.219	2.013	0.205	1.998	0.208
1000	0.556	0.484	1.823	0.716	1.975	0.572	1.450	0.558
	1.499	0.094	1.552	0.157	2.010	0.187	2.003	0.157
	2.042	0.188	1.979	0.182	2.013	0.148	1.971	0.172

**Table 5:** Descriptive statistics for the two data sets.

	*n*	Mean	Median	Standard Deviation	Skewness	Kurtosis
First data set	30	1.54	1.23	1.13	1.23	1.04
Second data set	179	89.13	51	105.56	2.21	5.64

**Table 6:** MLEs and their standard errors in parentheses for the first data set.

Model	*a*	*b*	θ	λ	α	β
TIKEx	40.5241	2.2765	0.0271	-	-	-
	(2.7618)	(0.6944)	(0.0446)	-	-	-
WEx	-	-	-	0.1013	8.4803	1.3238
	-	-	-	(0.0470)	(6.2815)	(0.1776)
LEx	69.2568	11.1652	0.0755	-	-	-
	(8.2345)	(6.1143)	(0.01261)	-	-
GEx	-	-	-	0.9649	0.3993	1.1085
	-	-	-	(0.4346)	(0.1894)	(0.5843)
BrEx	38.7695	0.0365	5.2164	-	-	-
	(9.9407)	(0.1128)	(1.2006)	-	-	-
BXEx	-	-	0.4261	0.2399	-	-
	-	-	(0.0852)	(0.0237)	-	-
Ex	-	-	0.6482	-	-	-
	-	-	(0.1183)	-	-	-
W2	-	-	-	-	66.0352	43.2275
	-	-	-	-	(4.44084)	(3.51546)
W3	-	-	0.0794	-	1.3441	1.7189
	-	-	(0.0622)	-	(0.2322)	(0.2767)
LN	-	-	-	-	0.1628	0.8014
	-	-	-	-	(0.1463)	(0.1033)

**Table 7:** Statistical measures for the first data set.

Model	$-\hat{\ell }$	AIC	BIC	W${}^{*}$	A${}^{*}$	KS	*p*-Value (KS)
TIKEx	39.6679	85.3358	89.5394	0.0174	0.1287	0.0631	0.9998
WEx	40.2139	86.4278	90.6314	0.0365	0.2729	0.0973	0.9388
LEx	40.3989	86.7978	91.0014	0.0353	0.2236	0.0864	0.9784
GEx	41.3196	88.6392	92.8428	0.0711	0.5068	0.1239	0.7459
BrEx	41.5335	89.0670	93.2706	0.0449	0.3360	0.1097	0.8631
BXEx	42.9933	89.9866	92.7890	0.1187	0.8107	0.1649	0.3877
Ex	43.5300	89.0600	90.4611	0.0189	0.1439	0.1845	0.2589
W2	43.1745	90.3491	93.1515	0.0183	0.1395	0.1889	0.2344
W3	39.7242	85.4484	89.6520	0.0246	0.1962	0.0979	0.9358
LN	40.7353	85.4707	88.2731	0.0391	0.2737	0.0970	0.9399

**Table 8:** Maximum likelihood methods (MLEs) and their standard errors in parentheses for the second data set.

Model	a	b	θ	λ	α	β
TIKEx	2.2601	1.2920	0.0073	-	-	-
	(1.0398)	(0.1740)	(0.0014)	-	-	-
WEx	-	-	-	2.9055	0.7768	0.0024
	-	-	-	(0.4138)	(0.0441)	(0.0002)
LEx	3.9060	1.0496	0.0108	-	-	-
	(4.5170)	(0.5452)	(0.0056)	-	-
GEx	-	-	-	0.7496	0.0044	0.7989
	-	-	-	(0.1327)	(0.0007)	(0.1188)
BrEx	0.0112	0.9459	0.9739	-	-	-
	(0.0024)	(0.1413)	(0.2126)	-	-	-
BXEx	-	-	0.4261	0.2399	-	-
	-	-	(9.5 × $10^{-5}$)	(.0017)	-	-
Ex	-	-	0.0112	-	-	-
	-	-	(0.0008)	-	-	-
W2	-	-	-	-	210.6370	3.2537
	-	-	-	-	(4.6020)	(0.9242)
W3	-	-	-	0.3540	0.9100	85.2728
	-	-	-	(0.3317)	(0.0364)	(6.5928)
LN	-	-	-	-	3.8393	1.2405
	-	-	-	-	(0.0927)	(0.0655)

**Table 9:** Statistical measures for the second data set.

Model	$-\hat{\ell }$	AIC	BIC	W${}^{*}$	A${}^{*}$	KS	*p*-Value (KS)
TIKEx	977.4752	1960.9500	1970.5130	0.0501	0.3418	0.0470	0.8237
WEx	985.8205	1977.6410	1987.2030	0.2669	1.6410	0.0755	0.2588
LEx	982.0106	1970.0210	1979.5830	0.1416	0.8632	0.0926	0.0927
GEx	997.0995	2000.1990	2009.7610	0.4779	2.9176	0.1279	0.0057
BrEx	982.1473	1970.2950	1979.8570	0.1918	1.1773	0.0746	0.2712
BXEx	1015.6880	2035.3750	2041.7500	0.7582	4.6283	0.1899	4.9 × $10^{-6}$
Ex	985.7355	1973.4710	1976.6583	0.1894	1.1638	0.0864	0.1380
W2	980.2322	1964.4640	1970.8390	0.0751	0.5026	0.0576	0.5910
W3	982.1508	1970.3020	1979.8640	0.1724	1.0704	0.05841	0.5745
LN	980.8582	1965.7164	1972.0911	0.0535	0.4329	0.0400	0.9360
